# 3D-printed N95 equivalent for personal protective equipment shortages: the Kansas City Mask

**DOI:** 10.2217/3dp-2020-0019

**Published:** 2021-02-04

**Authors:** Shiv Dalla, Brandon Bacon, Jack M Ayres, Stephen Holmstead, Alan J Ahlberg Elliot

**Affiliations:** 1 ^1^University of Kansas School of Medicine, Kansas City, KS 66160, USA; 2 ^2^Department of Surgery, University of Missouri, Kansas City, Truman Medical Center, Kansas City, MO 64108, USA; 3 ^3^Unaffiliated, Kansas City, KS 66160, USA

**Keywords:** 3D printers, 3D printing, COVID-19, infection control, N95, personal protective equipment, PPE, safety, stopgap PPE

## Abstract

Personal protective equipment (PPE) shortages represent a persistent and critical challenge during the COVID-19 pandemic. Communities of 3D printing hobbyists and experts responded by designing and producing homemade, 3D-printed PPE. This report discusses the design, manufacturing and validation of the Kansas City Mask (KC Mask). Once printed and assembled, masks were fit tested at Truman Medical Center in Kansas City, MO. The KC Mask was approved for use by pandemic response administration staff at the hospital. Fortunately, due to adequate PPE supply at the time of this publication, wide utilization of the KC mask has not been required. The authors endorse the KC Mask as a stopgap measure, proven to be effective in situations of critical PPE shortage based on Centers for Disease Control and Prevention (CDC) guidelines.

## Background

The novel coronavirus, named ‘SARS-CoV-2,’ represents a critical and serious threat to public health [1]. Highly contagious and virulent, the virus has been reported to cause severe respiratory problems in infected patients and was declared a pandemic by the WHO in March 2020 [1]. As the potential global economic impact of the pandemic came to light [2], the healthcare industry faced an overwhelming challenge: the adequate supply of appropriate personal protective equipment (PPE) [3].

### PPE shortages

The shortage of PPE is well reported and discussed, not only in the healthcare sector, but across all of society as the demands for PPE skyrocketed [3–5]. Unfortunately, as hospitalizations for COVID-19-related illness continue to increase, recent reports indicate the supply of PPE is persistently and significantly less than demand [6]. Even though the supply has risen since the beginning of the pandemic, experts predict that the supply is insufficient for predicted caseloads and it may take years in order for stockpiles to be fully replenished [7]. PPE shortages have been shown to contribute to viral spread within healthcare environments and in the general community [8]. This, of course, leads to increased societal harm and ultimately, preventable death. Of concern, these shortages are shown to be significantly greater in communities of color and low socio-economic status [9]. Additionally, PPE shortages have been shown to disproportionately affected women. Given that most PPE is designed and stocked for males, women are often left with ill-fitting masks, gloves and goggles [8].

### 3D-printed alternatives

To address and mitigate these arising issues, 3D printing experts and hobbyists, known as ‘makers’ identified the challenge posed by the pandemic and sought to create homemade masks, face shields, gowns and even nasopharyngeal swabs [10]. Makers with access to 3D printers were encouraged to produce stop-gap PPE that could be used in clinical environments. The widespread availability and cost-effective nature of 3D printing lead to an incredible phenomenon of collaboration among makers to develop various PPE products [11]. The flexibility and robust applications of 3D printers was leveraged into a uniquely useful tool. Face shields, face masks and strap adjustment devices became particularly popular products.

The mask presented, the Kansas City Mask (KC Mask), is one such product that was created from the maker community in partnership with local physicians and hospitals. This report discusses the design, manufacturing and validation of the KC Mask design and its usage in the COVID-19 pandemic as well as future usage as stopgap PPE.

## Materials & methods

### Mask design

The KC Mask is a stopgap, reusable, face mask substitute for level 1, level 3 or N95 face masks consisting of two 3D printed parts.

**Figure 1.**
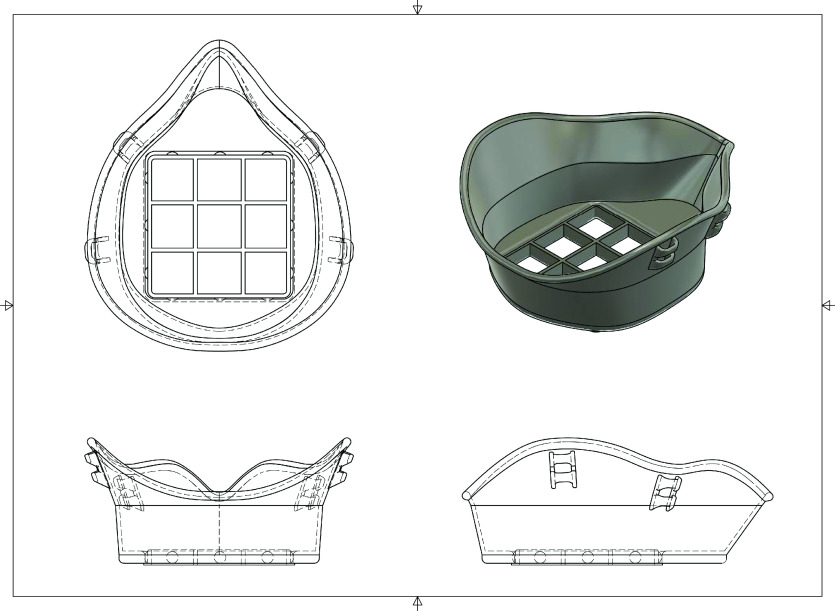
Multi view engineering drawing of Kansas City mask with filter holder in place.

As shown in Figure 1, the main component of the mask is the contoured surface that makes contact with the user’s face. On the external surface of the main component there is a 60 mm × 60 mm square hole where the second component, the filter holder, a 60 mm × 60 mm grid, will snap into place, holding in the space filter material. The mask accepts most fabrics as filter material. In this study, Halyard H600 sterilization wrap, which is commonly used in hospitals for packaging of sterilized surgical instruments and N99 rated, was used.

The Kansas City Mask was adapted from a similar design called the Montana Mask [12]. The goal of this redesign was to address some disadvantages of the Montana Mask [12], namely ease of breathing and fit. The Montana Mask has a smaller filter cross sectional area, making it more difficult to breath with higher grade filtration materials. KC Mask design is additionally enhanced by the dipping process explained in its respective section.

### Design & 3D printing

The design for the KC Mask was generated using commercial computer aided design (CAD) software and exported to an standard tessellation language (STL) file for compatibility with hobbyist 3D printer workflows. These workflows use a ‘slicer’ software to convert the universal STL file into GCODE that is specific to an individual 3D printer. Two combinations of slicer and printer were tested. The Cura slicer was used to prepare GCODE for a Lulzbot Taz5 and PrusaSlicer was used to prepare GCODE for a Creality CR-10S.

Masks were printed in a variety of filaments including polylactic acid (PLA), polyethylene terephthalate glycol (PET-G) and thermoplastic polyurethane (TPU). PLA was selected for further fit testing for its ease of use, cost–effectiveness and ability to be safely thermoformed to a user’s face in hot water. It is well known that fused deposition modeling of PLA can introduce porosity in a part’s exterior [13,14], which varies with model geometry and printer settings. Also, infill is a common practice in fused deposition modeling which intentionally introduces voids in the interior to conserve print time and material. Together, porosity and infill create the risk of liquid becoming trapped inside the part if it is submerged during sterilization. To mitigate this risk, we selected print settings such that the side walls were solid and only the flat section contained infill. To test for gross water permeability, a solid filter holder was printed and placed into the mask. Water was then poured into the inside of a mask. Over time, no water was shown to leak out, thus validating that there were no large filling defects in printing. Since quality control is difficult to maintain on hobbyist printers, we recommend qualitative fit testing (QLFT) before using any printed mask. For masks submitted to QLFT, the print settings shown in Table 1 were used.

**Table 1.** Print settings.

Selected print setting	Value
Layer height	0.25 mm
Wall line count	4
Top/bottom layers	4
Infill density	20%
Printing temperature (nozzle)	205°C
Printing temperature (build plate)	60°C

### Dipping & fitting

**Figure 2.**
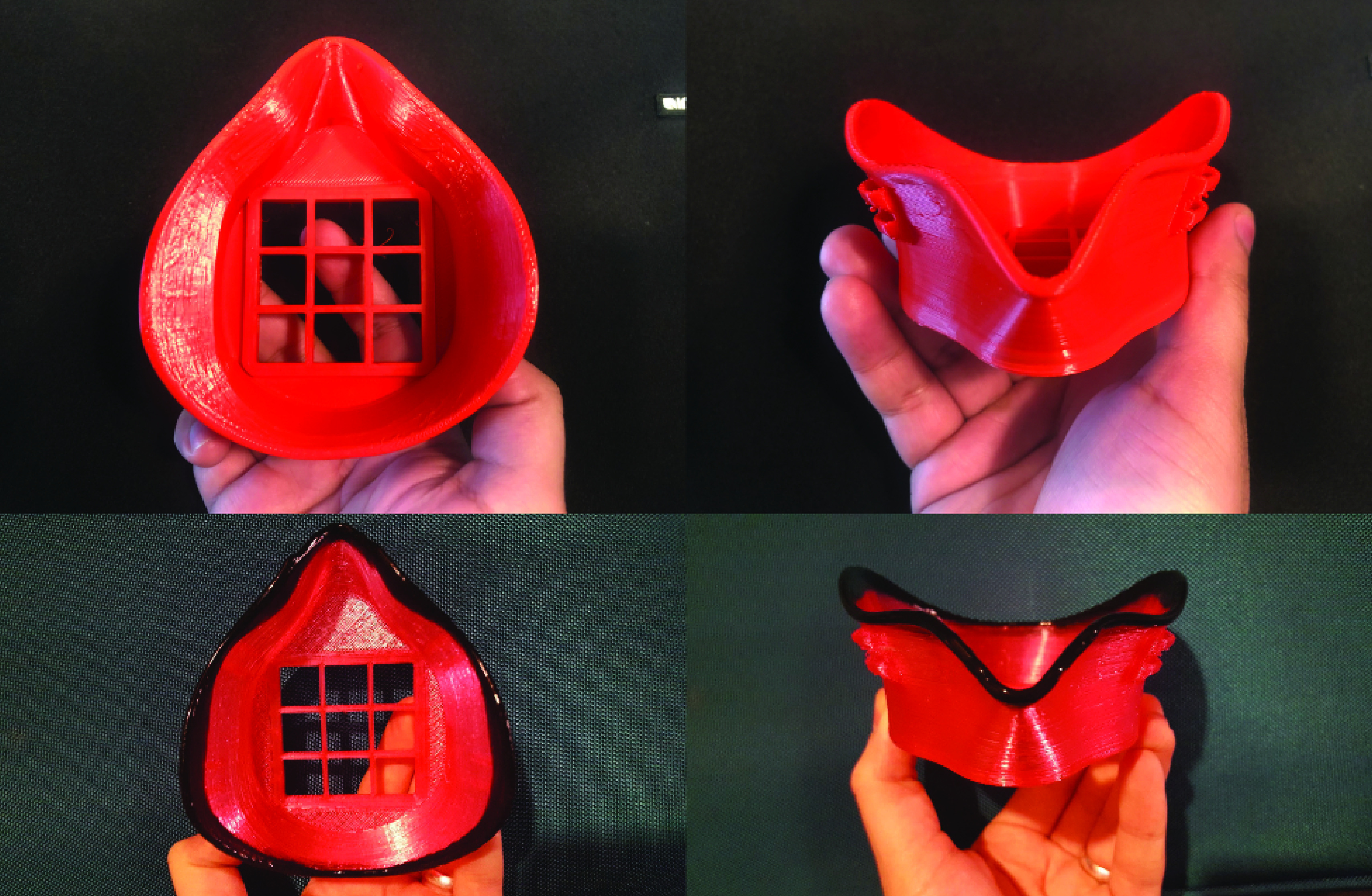
Stepwise images of mask following printing (top) and dipping (bottom) stages.

Dipping, shown in Figure 2, is an optional process in which liquid rubber is added to the rim of the mask which makes contact with the wearer’s face. This allows for a better seal as well as increased comfort. Flex Seal rubberized sealant was used in testing due to its lack of respiratory hazards. An optional dipping tray model is provided to help with this process. The goal is to coat the rim of the mask that will be touching the users face. Alternatively, weather stripping can be utilized for the same purpose, though this reduces ease of sterilization once applied. Weather stripping, commonly used in construction applications to ensure a water-tight seal of windows or doors, has a hollow or foam core and is therefore more porous than the Flex Seal rubberized sealant. Flex Seal, being less porous, is able to withstand being submerged in sterilizing liquid, such as dilute bleach, without absorption. Though other mask models utilize weather stripping, the KC Mask utilizes Flex Seal for the aforementioned reasons.

After the mask is dipped (Figure 2), it can be molded to the wearer’s face by increasing the temperature of the mask above the glass transition point of the material. For PLA, the easiest way to do this is to submerge the mask (including the filter holder, but without any filter material) in hot water (∼60°C). This allows for the PLA to become soft enough to mold, but the structure is maintained. The wearer should submerge the PLA for approximately 10 seconds and mold it to fit their face. This can be repeated until a good and comfortable fit is achieved.

### Fit testing

The KC Mask was fit tested at Truman Medical Center in Kansas City, MO, USA with the help of resident physician Dr Brandon Bacon and the facilities staff. Following Occupational Safety and Health Administration (OSHA) guidelines, a standard QLFT was performed using saccharin solution aerosol protocol [15]. First, a 3M 1860 N95 respirator was tested as a positive control. Then, the KC Mask, with Halyard 600 sterilization wrap as a filter, was fit tested using the same saccharin procedure. Although, quantitative fit testing (QNFT) was not performed, successful QLFT is recommended by OSHA [15] and has been shown to be very predictive of successful QNFT [16], making QLFT a reliable metric of a mask’s efficacy. Although, additional testing can and should be done, the authors believe the intensity and variety of testing performed was sufficient for the intent and purpose of this mask which is to be used as stopgap PPE during times of critical PPE/N95 shortages.

### Usage

**Figure 3.**
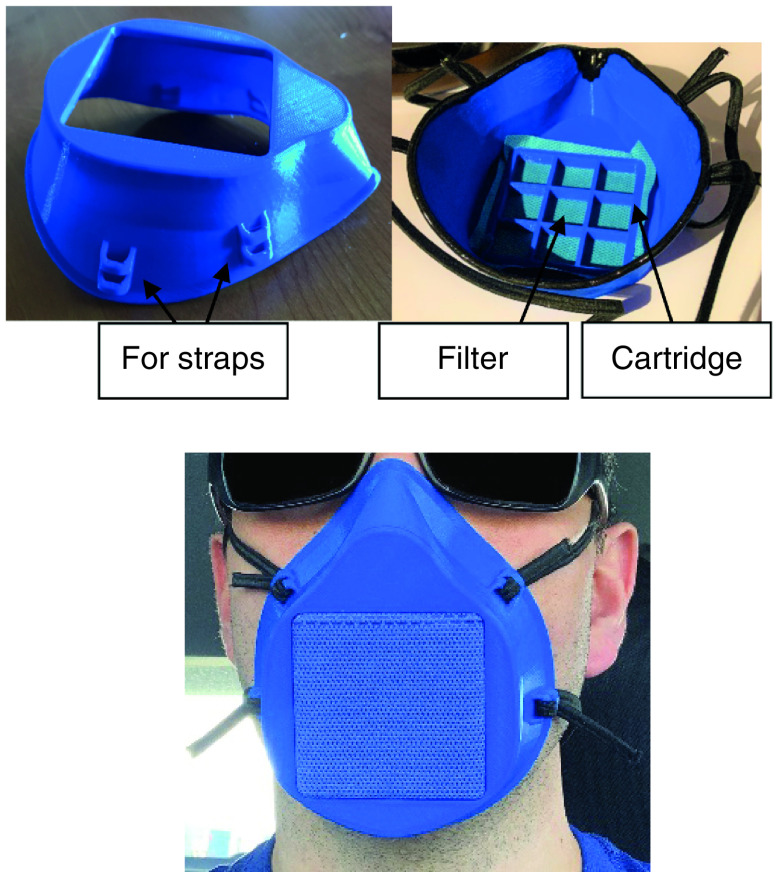
Assembly and usage instructions.

Elastic straps are used to secure the mask to the wearer’s face as demonstrated in Figure 3. Supplementary Figure 1 is an instruction sheet provided to users to explain assembly, including molding, placing straps and inserting the filter. The images below in Figure 3 are also included in the instruction sheet in Supplementary Figure 1. Sterilization of the mask is done using a Sani wipe or dilute bleach solution. Filters are meant to be used only once or can be re-sterilized per institutional protocol.

### Study design

The Institutional Review Board at Truman Medical Center/University of Missouri-Kansas City School of Medicine reviewed our proposal and determined that it was exempt from formal Institutional Review Board approval and was a proof of concept and quality improvement initiative. Although, further analysis and study to prove efficacy is required, the purpose of this study and report is to discuss the design, manufacturing and validation of the KC Mask concept.

## Results

Several dozen masks were distributed to Truman Medical Center. As a proof of concept, the mask was donned and a standard QLFT was performed. The QLFT was successful and the KC Mask was approved for usage by pandemic response administration staff at the hospital as a stop-gap measure in the event that standard N95 PPE became depleted. The KC Mask was not widely utilized, however, because Truman Medical Center maintained adequate PPE supply chains up to the time of this publication.

## Discussion

The results of Truman Medical Center’s approval of the KC Mask are promising for this N95 stopgap substitute. Although QLFT is less rigorous than QNFT, it has been shown to be highly correlated with proper mask seal and fit [16]. More extensive testing can and should be done, including QNFT and design modification.

In light of the current persistent PPE shortages, as well as potential shortages in the case of increasing caseloads, it is important to consider PPE and N95 alternatives, particularly those outside of the main supply chain of conventional PPE. This is critical for a number of reasons, the most obvious being stopgap PPE in healthcare settings in situations of critical shortages. Additionally, alternative PPE such as that described in this report can create access to PPE for additional groups without compromising current PPE supply channels. This could include providing N95 mask alternatives to individuals living with somebody who tests positive for COVID-19, a situation in which more readily available cloth or surgical masks would not be sufficient. This is especially important when considering that shortages of PPE are particularly common in minority communities and communities of low socio-economic status [9]. As stated by Brown Emergency Medicine (Rhode Island) Physician Dr Megan Ranney, “Whether it is the elderly, minority populations, populations affected by structural racism and historical injustice, those are the very groups that have the most difficult time getting adequate PPE for their workers” [9]. PPE shortages are largely seen as one of the biggest drivers of the racial divide in the COVID-19 pandemic in which infection rates and hospitalization rates disproportionately favor African American and Latino populations [9]. Accordingly, technology such as that which is presented in this report has the potential to uniquely help our country’s most vulnerable.

3D printed stopgap N95 masks similar to the KC Mask, such as the Montana Mask [12], have been designed, tested and validated for use in similar situations as the KC Mask. However, the KC Mask was created to address some of the disadvantages of these other designs as voiced by healthcare providers. The KC Mask has a lower profile, allowing for the simultaneous use of a face shield and/or eye protection. Given the importance of the face shield and eye protection as PPE in healthcare settings, it was imperative that the KC Mask was compatible with these other aspects of a provider’s PPE. Additionally, the KC Mask has a larger filter surface area allowing for improved ventilation for the wearer, reducing the effort of breathing. Other masks are ‘one size fits all’, which can be problematic for proper fitment and seal. A uniquely important advantage of the KC Mask is that once printed, the mask is specifically molded to the wearer’s face. The combination of the custom fit along with the Flex Seal coated rim allows for a comfortable and secure mask. Finally, many of the similar 3D printed masks require a multitude of parts that must be assembled. The KC Mask is only two 3D printed parts (the mask and filter holder), not including the dipping tray – which can be reused to dip as many masks as necessary. This lowers the burden on healthcare workers who may need to sterilize, don and doff the mask rapidly. The relatively small number of parts additionally makes for rapid manufacturing.

Although further analysis and study is needed for this design, persistently increasing caseloads and PPE shortages necessitates an urgent dissemination of these preliminary results. The authors do not advocate for the KC Mask as a replacement of traditional N95 masks or other PPE but do endorse the KC Mask as a stopgap measure, proven to be effective in situations of critical PPE shortage.

## Conclusion

The results of Truman Medical Center’s approval of the KC Mask are promising for this N95 alternative. Although further analysis and study is needed for this design, persistently increasing caseloads and PPE shortages necessitates an urgent dissemination of these preliminary results. The authors do not advocate for the KC Mask as a replacement of traditional N95 masks or other PPE but do endorse the KC Mask as a stopgap measure, proven to be effective in situations of dire PPE shortage.

**Summary points**

- As hospitalizations for COVID-19-related illness continue to increase, many recent reports indicate the supply of personal protective equipment (PPE) is persistently and significantly less than the demand.
- These PPE shortages encouraged communities of 3D printing experts and hobbyists to design and distribute homemade, 3D-printed PPE, including N95 mask substitutes. The mask presented, the Kansas City Mask (KC Mask), is one such product which was created from the maker community in partnership with local physicians and hospitals.
- The KC Mask was adapted from a similar design called the Montana Mask and created to address some of the disadvantages of other designs as voiced by healthcare providers:
  - Lower profile mask, allowing for the simultaneous use of a face shield and/or eye protection.
  - Larger filter surface area allowing improved ventilation for the wearer, reducing the effort of breathing.
  - Once printed, the mask is specifically molded to the wearer’s face. The combination of the custom fit along with the Flex Seal coated rim allows for a comfortable and secure mask.
  - The KC Mask is only two 3D printed parts (the mask and filter holder), resulting in lowers configuration burden on healthcare workers who may need to sterilize, don and doff the mask rapidly and frequently.
  - The relatively small number of parts additionally makes for rapid manufacturing.
- The design for the KC Mask was generated using commercial CAD software and exported to an STL file for compatibility with hobbyist 3D printer workflows.
  - Two combinations of slicer and printer were tested. The Cura slicer was used to prepare GCODE for a Lulzbot Taz5 and PrusaSlicer was used to prepare GCODE for a Creality CR-10S.
  - Polylactic acid was selected for further fit testing for its ease of use, cost–effectiveness and ability to be safely thermoformed to a user’s face with the use of hot water.
- Masks were fit tested by qualitative fit testing (QLFT) at Truman Medical Center in Kansas City, MO, USA as a proof of concept. The QLFT was successful and the KC Mask was approved for use by pandemic response administration staff at the hospital. Successful QLFT is a good predictor of a successful quantitative fit test.
- The authors do not advocate for the KC Mask as a replacement of traditional N95 masks or other PPE but do endorse the KC Mask as a stopgap measure, proven to be effective in situations of dire PPE shortage based on Centers for Disease Control and Prevention (CDC) guidelines.
