# Supplementary material for: 3D-printed N95 equivalent for personal protective equipment shortages: the Kansas City Mask
Source: J 3D Print Med. 2021 Feb 4:10.2217/3dp-2020-0019. doi: 10.2217/3dp-2020-0019 (PMC7860929; doi:10.2217/3dp-2020-0019)
Supplement: Supplementary file 1 [file supplementary_figure.pdf]

Kansas City 3D Printed Mask
Care Instructions

If PPE is not available, this mask is provided as a last resort. Use a certified medical N95 mask if available.

The mask is made of a plastic called polylactic acid (PLA). It is a rigid plastic that becomes malleable above 65 C.

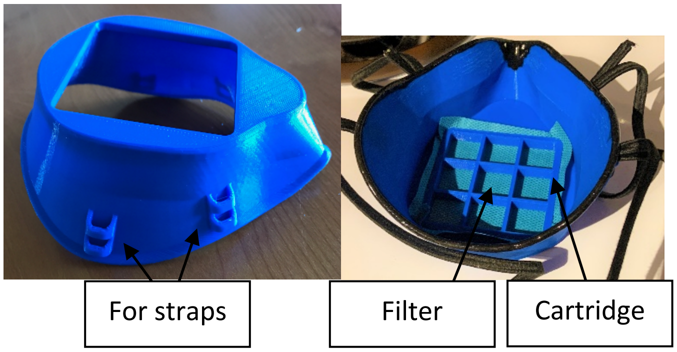

Preparing mask for use:
Before using your mask for the first time, sanitize the mask. This can be done with Orange top Sani-cloth wipes or a diluted solution of bleach*.

Mold it comfortably to your face:
1. Warm water to barely simmering ~65-70˚ celsius.
2. Keeping the filter cartridge in place, dip the edge of the mask (that sits on your face) in the water ~10 seconds.
3. Remove the mask from the water
4. Quickly wipe the water off the mask, apply it to your face, and mold the mask to your face
5. Repeat these steps until the mask makes a comfortable seal around your mouth and nose.

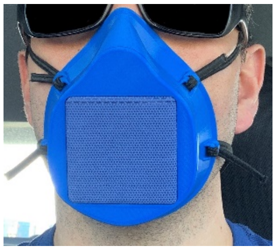

Place straps and adjust:
1. Use two 1/4"W elastic headbands (cut in half) OR two 18"L x 3/8"W elastic waist band material.
2. Insert the band into the strap connector from back to front (see picture)
3. Loop the band back through the space between the two connectors (see picture). This allows you to adjust the tightness once the mask is placed on your face
4. Repeat steps 1-3 on the other side
5. Repeat steps 1-5 for the second pair of strap connectors.

Make and insert filter:
1. Cut a 3"x3" square using a filter material. Examples: an N95 mask with broken elastics, sterile wrap from the OR, or any other filtration material available. Avoid materials with fiberglass as they are dangerous to inhale.
2. Lay the 3"x3" filter square and center the filter cartridge on the inside of the mask
3. Push the filter cartridge into place from inside the mask with the flange on the inside
4. Note: Push near the edges where the support struts are located to reduce risk of breaking the cartridge

After using the mask:
1. Wipe down all surfaces of the mask with an orange sani wipe or submerge in the bleach solution for 12-15 minutes after each day of use. The filter can be sterilized per local protocol or discarded and replaced.

CAUTION!
- The PLA material has a low melting point and will melt if left at high temperatures, such as in a car on a hot day. Protect your mask by keeping it indoors when it is hot outside and away from warm machinery.
- If your mask breaks, discard the mask and do not continue using it. If a seal cannot be created, then the mask will not be able to protect you.

***Bleach solution for sanitizing masks:**
All equipment that comes into contact with mucous membranes must receive either High Level Disinfection (HLD) or sterilization before use on another patient. HLD can be achieved by soaking in a dilute sodium hypochlorite solution for a minimum of 12 minutes. This solution can easily be prepared from commercially available household bleach, nearly always available as a 5.25% - 6.15% solution (52,500 ppm – 62,500 ppm).

The dilute solution for soaking this equipment should be prepared as a 1:20 dilution, mixing 1 part household bleach to 20 parts tap water, rendering approximately 2,500 ppm – 3,000 ppm concentration. Mix 200 ml of bleach (5.25% - 6.15% sodium hypochlorite) in 4 liters water. If the bleach is either more or less concentrated, alter the dilution accordingly to achieve the ppm noted above.

CAUTION: Hypochlorite solutions must not mix with ammonia solutions as the mixture can produce toxic chlorine gas. In mixing this solution, it is advised to err on the slightly more concentrated side (i.e., to greater than the minimum concentration needed) due to degradation of the hypochlorite that occurs over time, especially in open containers.

After soaking 12-30 minutes, the equipment should be rinsed with clean water and readied for use.

This is a modified version of the Montana Mask created by Dr. Dusty Richardson (MD), Dr. Spencer Zaugg (DDS) and Colten Zaugg.
Modifications made by Dr. Brandon Bacon (MD) with the engineering team at PPE for KC.
Basic questions about 3D printed masks can be found at MakeTheMasks.com
